# Space–time self-harm and suicide clusters in two cities in Taiwan

**DOI:** 10.1017/S2045796023000513

**Published:** 2023-06-01

**Authors:** Fang-Wen Lu, Erica Conway, Ya-Lun Liang, Ying-Yeh Chen, David Gunnell, Shu-Sen Chang

**Affiliations:** 1 Institute of Epidemiology and Preventive Medicine, College of Public Health, National Taiwan University, Taipei, Taiwan; 2 Institute of Health Behaviors and Community Sciences, College of Public Health, National Taiwan University, Taipei, Taiwan; 3 Global Health Program, College of Public Health, National Taiwan University, Taipei, Taiwan; 4 Taipei City Psychiatric Centre, Taipei City Hospital, Taipei, Taiwan; 5 Institute of Public Health and Department of Public Health, National Yang-Ming Chiao Tung University, Taipei, Taiwan; 6 Centre for Academic Mental Health, Population Health Sciences, University of Bristol, Bristol, UK; 7 National Institute of Health Research Biomedical Research Centre, University Hospitals Bristol and Weston National Health Service Foundation Trust, Bristol, UK; 8 Psychiatric Research Center, Wan Fang Hospital, Taipei Medical University, Taipei, Taiwan

**Keywords:** epidemiology, mental health, risk factors, scan statistics, self-harm, suicide, suicide cluster, Taiwan

## Abstract

**Aims:**

Suicidal acts may cluster in time and space and lead to community concerns about further imitative suicidal episodes. Although suicide clusters have been researched in previous studies, less is known about the clustering of non-fatal suicidal behaviour (self-harm). Furthermore, most previous studies used crude temporal and spatial information, e.g., numbers aggregated by month and residence area, for cluster detection analysis. This study aimed to (i) identify space–time clusters of self-harm and suicide using daily incidence data and exact address and (ii) investigate the characteristics of cluster-related suicidal acts.

**Methods:**

Data on emergency department presentations for self-harm and suicide deaths in Taipei City and New Taipei City, Taiwan, were used in this study. In all-age and age-specific analyses, self-harm and suicide clusters were identified using space–time permutation scan statistics. A cut-off of 0.10 for the *p* value was used to identify possible clusters. Logistic regression was used to investigate the characteristics associated with cluster-related episodes.

**Results:**

A total of 5,291 self-harm episodes and 1,406 suicides in Taipei City (2004–2006) and 20,531 self-harm episodes and 2,329 suicides in New Taipei City (2012–2016) were included in the analysis. In the two cities, two self-harm clusters (*n* [number of self-harm episodes or suicide deaths in the cluster] = 4 and 8 in Taipei City), four suicide clusters (*n* = 3 in Taipei City and *n* = 4, 11 and 4 in New Taipei City) and two self-harm and suicide combined clusters (*n* = 4 in Taipei City and *n* = 8 in New Taipei City) were identified. Space–time clusters of self-harm, suicide, and self-harm and suicide combined accounted for 0.05%, 0.59%, and 0.08% of the respective groups of suicidal acts. Cluster-related episodes of self-harm and suicide were more likely to be male (adjusted odds ratio [aOR] = 2.22, 95% confidence interval [CI] 1.26, 3.89) and young people aged 10–29 years (aOR = 2.72, 95% CI 1.43, 5.21) than their cluster-unrelated counterparts.

**Conclusions:**

Space–time clusters of self-harm, suicide, and self-harm and suicide combined accounted for a relatively small proportion of suicidal acts and were associated with some sex/age characteristics. Focusing on suicide deaths alone may underestimate the size of some clusters and/or lead to some clusters being overlooked. Future research could consider combining self-harm and suicide data and use social connection information to investigate possible clusters of suicidal acts.

## Introduction

A space–time suicide cluster can be defined as multiple suicides that occur closer to each other in time and space than would be expected based on the expectation of the community or statistical chance (Robinson *et al.*, 2016a). One single suicide cluster could have a tremendous impact on the community and lead to serious concerns of imitation, i.e., further occurrences of suicide (Heffel *et al.*, 2015; Robinson *et al.*, 2016a, 2016b).

Previous studies of clusters of suicidal behaviour have mainly focused on suicide deaths (Benson *et al.*, 2022; Haw *et al.*, 2013; Niedzwiedz *et al.*, 2014; Robinson *et al.*, 2016a), although non-fatal suicidal behaviour may also occur as part of a cluster of suicide deaths, and the incidence of non-fatal suicidal behaviour can be 20-fold higher than that of fatal events (Fazel and Runeson, 2020). Not including non-fatal suicidal behaviour in the analysis may lead to many clusters of suicidal behaviour being overlooked.

Two previous studies have investigated clusters of suicidal behaviour by including both suicide deaths and non-fatal suicide attempts. Too *et al.* (2017) analysed the spatio-temporal clusters of (i) hospital admissions for suicide attempt and (ii) suicide deaths separately using data from Western Australia. The study identified a small proportion of suicide attempts (1%) and suicides (0.6%) occurring within clusters, and the clusters of suicide attempts and suicides were geographically close to each other. The same study also found an association of cluster-related suicide attempts with several area characteristics such as low socioeconomic status, the proportion of people who had changed addresses in the previous year, and the proportion of indigenous people. Another study by Too *et al.* (2019) focused on young people aged 15–24 years and used data for suicidal acts (suicide attempts and suicides combined) from Western Australia and New South Wales for two periods. The study showed that cluster-related suicidal acts accounted for 0–3% of all events over the periods studied.

The two previous studies used aggregated data for suicide attempt and suicide by the month of occurrence and geographic area, which has an average population size of 10,000–16,000; the analysis used area centroids as a proxy for the person’s place of residence (Too *et al.*, 2019, 2017). Calculating risks based on artificially defined areas might result in the modifiable areal unit problem, which means that the choice of aggregation area unit might influence the results of statistical tests and cause biases (Openshaw, 1984). By contrast, a scan statistic model that utilizes the exact location information (i.e., point data), such as Space Time Permutation Scan Statistics (STPSS), is available but was rarely used in past research to detect the clusters of suicidal behaviour, with only two exceptions (Jones *et al.*, 2013; Perez-Costillas *et al.*, 2015). Moreover, the two Australian studies used hospital admission data for suicide attempt, not including emergency department presentation data, and the residential address information was missing for a significant proportion of suicide attempts and suicides (up to 22–33%) – both could result in potential under-detection of suicidal behaviour clusters.

This study aimed to investigate the space–time cluster of suicidal acts (self-harm, suicides, and both combined) in two cities in Taiwan. Specifically, we examined (i) the prevalence of space–time clusters of different suicidal acts (self-harm, suicide, and self-harm and suicide combined) and (ii) the characteristics of cluster-related suicidal acts compared with those not in clusters. To bridge the gaps in previous literature, this study included emergency room presentations of self-harm and used data with fine-grained spatial information. We used the term ‘self-harm’ when referring to self-injury or self-poisoning, irrespective of the motivation and degree of suicidal intent (Hawton *et al.*, 2003).

## Method

### Data

**Figure 1.**
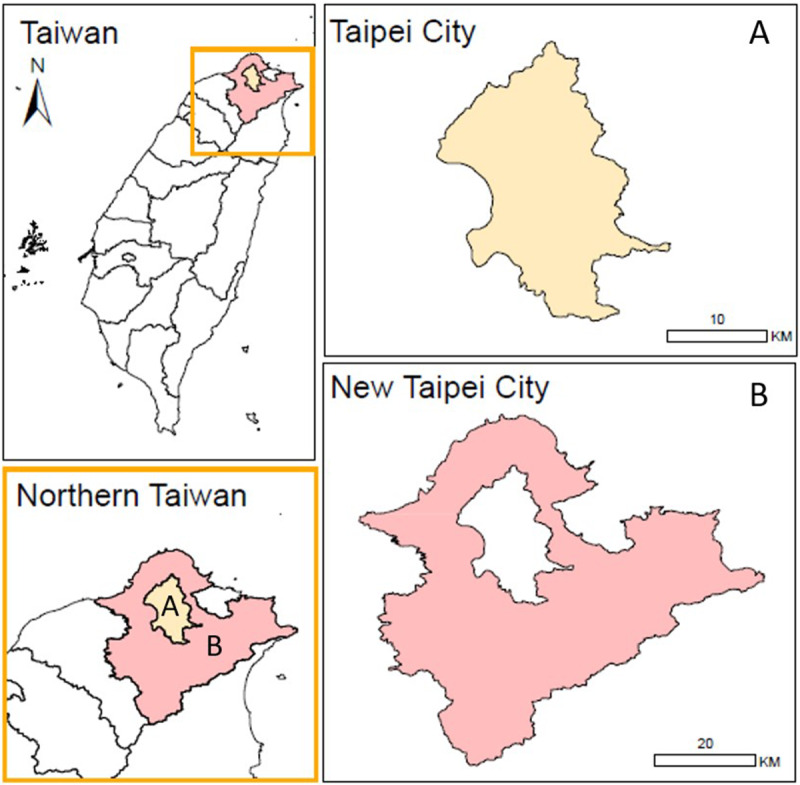
Location of the study cities: Taipei City (A) and New Taipei City (B) in Taiwan.

Taipei City (population = 2.6 million in 2004) and New Taipei City (population = 3.9 million in 2012) are both in northern Taiwan (Fig. 1). New Taipei City is the surrounding area of Taipei City, while it is officially a city of its own; both cities are part of the Taipei Metropolitan area. Self-harm and suicide data for Taipei City (2004–2006) and New Taipei City (2012–2016) were from two separate research projects supported by respective city governments.

Self-harm data for Taipei City were obtained from the city’s self-harm surveillance system. Details of the surveillance system were described elsewhere (Kuo *et al.*, 2012; Kwok *et al.*, 2014). In brief, all of the 26 hospitals in Taipei City were required to report self-harm presentations to emergency departments by uploading a form with the patients’ basic demographic information, current address and the date and method of self-harm onto the system, so that the aftercare workers can contact the patients and provide aftercare.

Suicides for Taipei City were identified from the national cause-of-death data files using the following International Classification of Diseases, Tenth Revision, (ICD-10) codes: X60-X84 for certified suicides; Y10-Y34 for undetermined deaths; X48 for accidental pesticide poisoning and W75, W76, W83 and W84 for accidental suffocation (Lin *et al.*, 2019). In keeping with findings from other countries (Gunnell *et al.*, 2013), previous studies from Taiwan (Chang *et al.*, 2010) showed that many of these undetermined and accidental deaths were likely to be misclassified suicides and were therefore included in the study. For simplicity, we used the term ‘suicide’ when referring to both certified and possible suicides combined in the paper.

Self-harm and suicide data for New Taipei City were obtained from a project investigating the geographic distribution of self-harm and suicide in the city (Liang, 2019) based on data from Taiwan’s National Suicide Surveillance System (NSSS), which included data for self-harm episodes, suicides and suicidal ideation presenting to emergency departments, the police, firefighters, or other governmental agencies (Chen *et al.*, 2016; Pan *et al.*, 2013). Patients’ basic demographic information, contact address, and the date and method of the suicidal acts were uploaded onto the system. Similar to Taipei City, suicide data for New Taipei City were extracted from the national cause-of-death data files to capture suicides not reported to the NSSS as well as possible suicides (Chang *et al.*, 2010).

Supplementary Figure S1 shows the flowcharts to identify self-harm episodes and suicides for the study. A total of 5,291 (89% of eligible episodes) and 20,531 (98%) self-harm records and 1,406 (99% of eligible cases) and 2,329 (95%) suicides in Taipei City and New Taipei City, respectively, were included in the analysis. The process to identify eligible self-harm episodes and suicides was described in detail in the Supplementary materials.

The Taiwan Geospatial One Stop (https://www.tgos.tw/tgos/Web/Address/TGOS_Address.aspx) was used to identify the geographic coordinates (latitudes and longitudes) for each suicidal episode based on the residential address. By adopting the coordinates of the exact address (i.e., point data) rather than using the centroid of its administrative district, the cluster identification results would not suffer from aggregation and therefore cluster location accuracy was kept for precise identification. When comparing the area-level characteristics between cluster-related and unrelated episodes of self-harm and suicide, each suicide or self-harm episode was assigned to one of the ‘neighbourhoods’ (an administrative unit; *n* = 432 in Taiwan City and 1,032 in New Taipei City) based on the geocoded coordinates.

Population data at the neighbourhood level for both cities were extracted from the household registration data. Data for median household income were extracted from the Income Tax Statistics.

### Statistical analysis

STPSS (Kulldorff *et al.*, 2005) was used to identify spatio-temporal clusters of self-harm episodes, suicides, and both combined in the two study cities over the respective time periods. The STPSS was adopted as this approach allows the use of point data (i.e., geographic coordinates of address) to identify clusters with more exact location information. STPSS uses a Poisson-based likelihood to identify clustering within a scanning window by assessing whether the number of observed events occurring inside the window is greater than that expected (Kulldorff *et al.*, 2005). The window is a cylinder with different radiuses and heights, which, respectively, indicate the cluster area and duration. The window that has the maximum likelihood ratio between the observed and expected numbers of cases is considered the most likely candidate for a space–time cluster. The temporal information used in the analysis was the day of the suicidal acts, and the spatial information was the geographic coordinates of the addresses. The maximum spatial and temporal size of the cluster was set at 10% of the population at risk and 1 year, respectively, based on previous research (Jones *et al.*, 2013; Milner *et al.*, 2018; Sugg *et al.*, 2021; Too *et al.*, 2017). Overlapping of clusters was allowed if less significant clusters did not centre in more likely clusters. The Monte Carlo method with 999 replications was used to generate *p*-values. Clusters with a *p* value less than 0.10 were considered as possible clusters in the study. SaTScan version 9.6 was used to conduct the spatio-temporal cluster detection analyses (Kulldorff, 2018).

All-age and age-specific analyses were conducted. Consistent with previous research in an Asian population (Yamaoka *et al.*, 2020), six different age groups were considered (10–19, 20–29, 30–39, 40–49, 50–59, and 60 years and above).

We conducted a robustness test to examine the impact of self-harm repetition. We intended to identify space–time clustering of suicidal acts of different individuals rather than repeated episodes by the same individuals. Similar to the approach used by Too *et al.* (2017), a two-stage analysis was conducted. In the first stage, all suicidal acts, including repeated self-harm episodes, were included in the space–time cluster detection analysis. If any detected clusters contained repeated self-harm episodes by the same individuals, one of the episodes was randomly chosen, with the others being excluded, and a cluster detection analysis was re-run to check for evidence of a similar spatio-temporal cluster. The re-run was repeated multiple times according to the maximum number of individual repetitions in the target cluster. A cluster passed the test if a similar cluster covered more than 50% of the space–time period and individuals of the target cluster in any of the re-runs. Final cluster detection results were plotted using ArcGIS 10.7.

The cluster detection analysis revealed some groups of suicides having the same address and date of occurrence. These are likely to be ‘suicide pacts’, i.e., where several people agree to take their lives together at the same time. News reports of suicides were searched and descriptions were compared with these possible suicide pacts regarding sex, age, date of death, and address to determine if these were actually suicide pacts. The etiology of suicide pacts would differ from suicide clusters, and thus they were not included in the analysis of the characteristics of cluster-related vs cluster-unrelated episodes.

Logistic regression analysis was conducted to investigate the characteristics associated with cluster-related episodes of self-harm and suicide. The outcome was a binary variable indicating whether the suicidal episode was in an identified cluster. For individuals with multiple self-harm episodes, we included only one episode for cluster-related cases and one episode for cluster-unrelated episodes, both chosen randomly. We, therefore, excluded a total of 893 self-harm repetition episodes (13.3% of 5,291 self-harm episodes and 1,406 suicides combined) in Taipei City and 5,376 self-harm repetition episodes (23.5% of 20,531 self-harm episodes and 2,329 suicides combined) in New Taipei City from the analysis. Individual-level characteristics included sex and age (10–29 years vs 30+ years), while area-level characteristics include neighbourhood population density and median household income (in terciles). Both unadjusted and sex/age-adjusted odds ratios (ORs) and their 95% confidence intervals (CIs) were estimated. We accounted for within-neighbourhood correlation using generalized estimating equations. The logistic regression analysis was conducted using the geepack package in R software (version 4.0.3).

## Results

Table 1 shows the number and rate of self-harm and suicide, as well as the ratio between the number of self-harm episodes (repeated episodes included) and suicide, overall and by sex and age in the two cities over the respective study periods. During 2004–2006, there were a total of 5,291 (male = 1,361; 26%) self-harm episodes and 1,406 (male = 939; 67%) suicides among people aged 10+ years in Taipei City (Table 1a). During 2012–2016, there were a total of 20,531 (male = 6,579; 32%) self-harm episodes and 2,329 (male = 1,465; 63%) suicides among people aged 10+ years in New Taipei City (Table 1b). The annual age-standardized self-harm and suicide rates (per 100,000) were 75.4 and 20.1, respectively, in Taipei City; the corresponding rates were 113.4 and 12.9, respectively, in New Taipei City in the study periods.

**Table 1.** The number and annual rate (per 100,000) of self-harm episodes and suicides in Taipei City (a) and New Taipei City (b), Taiwan

	Self-harm episodes		Suicides		
	*n*	Rate (per 100,000)	*n*	Rate (per 100,000)	Ratio of self-harm episodes to suicides
*(a) Taipei City (2004−2006)*					
Taipei City					
Total	5291	75.4	1406	20.1	3.8
Male	1361	39.9	939	27.6	1.5
Female	3930	109.0	467	13.0	8.4
Aged 10−19 years	370	36.7	23	2.3	16.1
Aged 20−29 years	1750	150.2	171	14.7	10.2
Aged 30−39 years	1306	104.5	250	20.0	5.2
Aged 40−49 years	964	70.5	341	24.9	2.8
Aged 50−59 years	462	44.0	258	24.6	1.8
Aged 60+ years	439	37.4	363	31.0	1.2
*(b) New Taipei City (2012−2016)*					
New Taipei City					
Total	20,531	113.4	2329	12.9	8.8
Male	6579	74.2	1465	16.5	4.5
Female	13,952	151.1	864	9.4	16.1
Aged 10−19 years	1152	50.6	43	1.9	26.8
Aged 20−29 years	4095	144.7	245	8.7	16.7
Aged 30−39 years	5823	167.9	472	13.6	12.3
Aged 40−49 years	4317	135.8	489	15.4	8.8
Aged 50−59 years	2739	86.9	422	13.4	6.5
Aged 60+	2405	75.3	658	20.6	3.7

In both cities, self-harm rates were higher in females than males, while suicide rates were higher in males than females. Self-harm rates were higher in younger groups (highest in 20- to 29-year-olds in Taipei City and 30- to 39-year-olds in New Taipei City); by contrast, suicide rates were highest in the oldest group aged 60+ years. The ratio between self-harm and suicide was highest in the youngest group aged 10–19 and decreased with age.

### Cluster detection using space–time permutation scan statistics

Table 2 shows a summary of identified clusters. Two self-harm clusters (Taipei City), four suicide clusters (one in Taipei City and three in New Taipei City) and two self-harm and suicide combined clusters (one in Taipei City and one in New Taipei City) were identified. These identified clusters all passed the robustness test. Additionally, three suicide pacts (one in Taipei City and two in New Taipei City) were also found.

**Table 2.** Self-harm and suicide space–time clusters in Taipei City (a) and New Taipei City (b), Taiwan

Cluster	Age group	Radius (km)	Start date (m/d/y)	End date (m/d/y)	Period in days	Observed*	Expected**	Observed/Expected	*p* value
*(a) Taipei City (2004−2006)*									
Self-harm									
1	10−19	0.3	3/3/2006	3/6/2006	4	4	0.08	49.33	0.025***
2	20−29	0.91	6/6/2005	6/14/2005	9	8	0.45	17.95	0.052
Suicide									
P_1_	10+	0	-/-/2004	-/-/2004	1	3	0.01	234.33	0.098
P_1_	40−49	0	-/-/2004	-/-/2004	1	3	0.03	113.67	0.012***
3	50−59	0.75	3/10/2006	3/11/2006	2	3	0.06	51.60	0.067
Self-harm and suicide									
P_1_	10+	0	-/-/2004	-/-/2004	1	3	<0.01	744.11	0.057
4^a^	10−19	0.3	3/3/2006	3/6/2006	4	4	0.08	52.40	0.005***
5^b^	20−29	0.91	6/6/2005	6/14/2005	9	8	0.44	18.30	0.014***
P_1_	40−49	0	-/-/2004	-/-/2004	1	3	0.01	435.00	0.012***
6^c^	60+	0.52	7/6/2005	7/11/2005	6	4	0.07	57.29	0.080
*(b) New Taipei City (2012−2016)*									
Suicide									
P_A_	10+	0	-/-/2012	-/-/2012	1	4	0.01	388.17	<0.001***
A	20−29	0.58	8/20/2013	9/21/2013	33	4	0.10	39.2	0.028***
B	40−49	8.55	2/28/2016	6/8/2016	102	11	1.60	6.87	0.056
C	40−49	0.66	8/11/2015	8/16/2015	6	4	0.08	48.9	0.077
P_B_	50−59	0.16	-/-/2016	-/-/2016	2	3	0.02	140.67	0.033***
Self-harm and suicide									
P_A_	10+	0	-/-/2012	-/-/2012	1	4	<0.01	879.23	0.002***
D^d^	20−29	0.74	9/15/2013	9/21/2013	7	8	0.35	22.84	0.038***

* The number of self-harm episodes or suicides in the cluster detected.

** The number of self-harm episodes or suicides that would have been expected if there was no cluster.

*** Clusters with a *p* value smaller than 0.05

a This cluster was the same as cluster 1 with four self-harm episodes.

b This cluster was the same as cluster 2 with eight self-harm episodes.

c This cluster was a combined cluster with two self-harm episodes and two suicides.

d This cluster included five self-harm episodes and three suicide deaths; the three suicide deaths were also included in cluster A, which contained four suicide deaths.

P_1_ was a suicide pact of three family members, with two by hanging and one by self-poisoning. The exact start and end dates of the suicide pact were removed to prevent individuals from being identified. The suicide pact was identified in the all-age (aged 10+ years) and age-specific (aged 40-49 years) analyses of suicides as well as suicides and self-harm episodes combined.

P_A_ was a suicide pact consisting of four family members who died by charcoal burning in a car. The exact start and end dates of the suicide pact were removed to prevent individuals from being identified. The suicide pact was identified in the all-age (aged 10+ years) analyses of suicides as well as suicides and self-harm episodes combined.

P_B_ included three suicides; of them, two individuals died by charcoal burning in a car (i.e., a suicide pact). The exact start and end dates of the suicide pact were removed to prevent individuals from being identified.

In Taipei City (Table 2a), the two self-harm clusters were identified in the age groups 10–19 (cluster 1, four events, *p* = 0.025; 1.08% of all self-harm events in this age group) and 20–29 years (cluster 2, eight events, *p* = 0.052; 0.46% of all self-harm events in this age group). The suicide cluster was identified in the age group 50–59 years (cluster 3, three events, *p* = 0.067; 1.16% of all suicides in this age group). Three clusters were identified when the self-harm and suicide data were combined; of them, two (clusters 4 and 5) were the same as clusters 1 and 2, respectively. The additional cluster was identified in the age group 60+ years (cluster 6, two self-harm episodes and two suicides, *p* = 0.080; 0.50% of all self-harm and suicide events in this age group).

In New Taipei City (Table 2b), no self-harm clusters were identified. Three suicide clusters were identified in the age groups 20–29 (cluster A, four events, *p* = 0.028; 1.6% of all suicides in this age group), 40–49 (cluster B, 11 events, *p* = 0.056; 2.2% of all suicides in this age group) and 40–49 years (cluster C, four events, *p* = 0.077; 0.8% of all suicides in this age group). One cluster containing five self-harm events and three suicides was identified in the age group 20–29 (cluster D, *p* = 0.038; 0.2% of all self-harm and suicide events in this age group); this cluster contained three of the suicides in suicide cluster A.

The clusters in both cities mostly occurred over a short time period (2–33 days) with a radius of less than 1 km, with only one exception spanning 102 days with a radius of 8.55 km in New Taipei City (Table 2). The number of events included in each cluster accounted for only a small percentage of all suicidal acts. Overall, self-harm events, suicides, and self-harm and suicides combined in the identified clusters (excluding suicide pacts) accounted for only 0.05% (12/25,822), 0.59% (22/3,735), and 0.08% (24/29,557) of the total number of events in the three groups, respectively.

**Figure 2.**
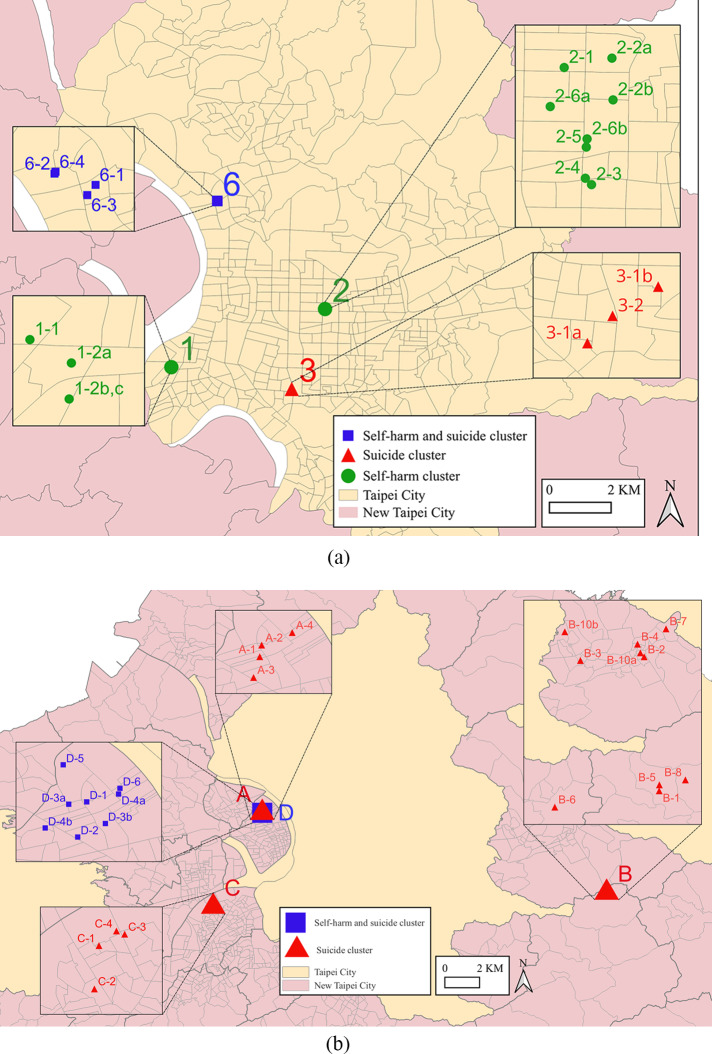
Self-harm and suicide clusters identified in Taipei City (a) and New Taipei City (b), Taiwan. See Table 2a and b for detailed data for these clusters.

Figure 2 shows the geographical distribution of the identified clusters. In Taipei City, the identified clusters were located in the west and southwest areas. In New Taipei City, clusters A (suicide only) and D (self-harm and suicide combined) were close to each other geographically.

(a) Taipei City (2004–2006) *Clusters 4 and 5 were not included in this map because they were the same as clusters 1 and 2, respectively. **The serial numbers assigned to the self-harm episodes or suicides in the insets were based on the chronological order of occurrence, determined by the dates on which each individual self-harm episode or suicide took place. For example, the 1st self-harm episode in the 1st self-harm cluster was labelled as ‘1-1’. For two or more self-harm episodes or suicides that occurred on the same day, they were given an additional letter, for example, the 2nd, 3rd and 4th self-harm episodes, which all occurred on the same day, of the 1st self-harm cluster were labelled as ‘1-2a’, ‘1-2b’ and ‘1-3c’, respectively. ***Self-harm episodes 1-2b and 1-2c occurred at the same address on the same day. This could be a possible self-harm pact, but it could not be confirmed due to a lack of detailed information. (b) New Taipei City (2012–2016) *Cluster D included five self-harm episodes and three suicides; the three suicides were also included in cluster A, which contained four suicides. **The serial numbers assigned to the self-harm episodes or suicides in the insets were based on the chronological order of occurrence, determined by the dates on which each individual self-harm episode or suicide took place. For example, the 1st suicide in suicide cluster A was labelled as ‘A-1’. For two or more self-harm episodes or suicides that occurred on the same day, they were given an additional letter. For example, the 10th and 11th suicides of the suicide cluster B occurred on the same day and were labelled as ‘B10a’ and ‘B10b’, respectively.

### Suicide pacts

A total of three suicide pacts were identified in the cluster detection analysis. One suicide pact (suicide pact P_1_, with three suicides) was identified in Taipei City (Table 2a). A search of news reports revealed that the suicide pact included three family members (two brothers and one sister aged between 43 and 49 years) who died by hanging (*n* = 2) and self-poisoning (*n* = 1), respectively, in the same address. The pact was consistently identified in the all-age (i.e., aged 10+ years) and age-specific (i.e., aged 40-49 years) analyses of suicides as well as suicides and self-harm episodes combined. Two suicide pacts (suicide pact P_A_, with four suicides, and suicide pact P_B_, with three suicides) were identified in New Taipei City (Table 2b). A search of news reports revealed that suicide pact P_A_ consisted of four family members (three adults and one adolescent) who died from carbon monoxide poisoning by burning barbecue charcoal in a car. The three suicides in suicide pact P_B_ included one couple (*n* = 2) who died together by burning charcoal in a car, i.e., a suicide pact, while the third suicide was unrelated to the couple and occurred on the next day. A sensitivity analysis that counted the couple as one single suicide event showed no more statistical evidence of this suicide cluster.

### Characteristics of cluster-related vs unrelated self-harms episodes and suicides

Based on combined data from Taipei City and New Taipei City, people who self-harmed or died by suicide as part of a cluster (*n* = 19 in Taipei City and *n* = 24 in New Taipei City, excluding suicide pacts) were more likely to be male (vs female) and younger (aged 10–29 vs 30+years) than their cluster-unrelated counterparts (Table 3). Sex/age-adjusted ORs were 2.22 (95% CI 1.26, 3.89) for males (reference group: females) and 2.72 (95% CI 1.43, 5.21) for 10- to 29-year-olds (reference group: individuals aged 30+ years). No association was found between cluster-related self-harm episodes and suicides and neighbourhood median household income or population density.

**Table 3.** Comparison of individual- and area-level characteristics between cluster-related and unrelated self-harm episodes and suicides in Taipei City and New Taipei City, Taiwan

	Cluster-related		Cluster-unrelated		Unadjusted			Adjusted for sex and/or age		
	*n*	(%)	*n*	(%)	OR	(95% CI)	*p* value	OR	(95% CI)	*p* value
Total	43	(100)	23,245	(100)						
Sex										
Female (ref)	19	(44)	14,684	(63)	1.00			1.00		
Male	24	(56)	8561	(37)	2.09	(1.19, 3.68)	0.011	2.22	(1.26, 3.89)	0.005
Age (years)										
30+ (ref)	22	(51)	17,052	(73)	1.00			1.00		
10−29	21	(49)	6193	(27)	2.55	(1.32, 4.91)	0.005	2.72	(1.43, 5.21)	0.002
Median household income										
Low (ref)	15	(35)	6342	(27)	1.00			1.00		
Medium	10	(23)	8476	(36)	0.49	(0.20, 1.18)	0.11	0.48	(0.20, 1.16)	0.10
High	18	(42)	8427	(36)	0.91	(0.39, 2.12)	0.82	0.92	(0.40, 2.14)	0.85
Population density										
Low (ref)	9	(21)	6259	(27)	1.00			1.00		
Medium	20	(47)	8773	(38)	1.51	(0.60, 3.78)	0.38	1.52	(0.61, 3.81)	0.37
High	14	(33)	8213	(35)	1.05	(0.41, 2.69)	0.92	1.12	(0.44, 2.86)	0.82

OR = odds ratio; CI = confidence interval.

## Discussion

Space–time clusters of suicidal acts were relatively rare; self-harm, suicide, and self-harm and suicide combined accounted for 0.05%, 0.59%, and 0.08% of the respective groups of suicidal acts. The analyses using combined data for non-fatal events (self-harm episodes) and fatal events (suicides) identified additional clusters compared with analyses based on self-harm or suicide data alone. People who self-harmed or died by suicide as part of a cluster were more likely to be male and younger than cluster-unrelated cases.

### Strengths and limitations

This is the first study to identify space–time clusters of self-harm, suicide, and self-harm and suicide combined using fine-grained event date and residential address information. The availability of point data could achieve high geographical accuracy by allowing cluster identification using the space–time permutation model, which is more specific than the discrete Poisson model based on aggregate area data used in previous studies. The data used in our analysis were comprehensive; around 90% or more of the eligible episodes had complete residential address information and were included in the analysis.

There are limitations to this study. First, we only considered the proximity of space (based on residence) and time (based on the date of self-harm or suicide) between individuals and could not analyse social network connections (Hawton *et al.*, 2020). Recent studies used data from the police and coroner reports to investigate the social links between cluster-related suicides (Hill *et al.*, 2020a, 2020b) or interviews with people who self-harmed to investigate the link between self-harm in these individuals and previous cluster-related suicides (John *et al.*, 2022). Second, our analysis comparing cluster-related and unrelated cases did not include information on other risk factors of suicidal behaviour such as drug or alcohol abuse and history of self-harm, which may contribute to the clustering of suicidal behaviour (Haw *et al.*, 2013). Third, the analysis of cluster characteristics had limited statistical power due to the small number of cluster-related self-harm episodes and suicides being identified. Fourth, the data covered two distinct periods in Taipei City and New Taipei City because of data availability. Furthermore, Taipei City had a lower ratio of self-harm episodes to suicides than New Taipei City; this may indicate that self-harm episodes could be under-detected in Taipei City, and this could impact cluster detection. Lastly, the space–time permutation model did not adjust for population number. If there was a large change in the population number within a short period of time in an area, in contrast to constant population in other areas, this might lead to false negative or positive results of cluster detection (Kulldorff, 2018). However, in our analysis, we set the maximum temporal size of the cluster at 1 year, and this would limit the impact of change in the population number over time, if any. Furthermore, our data showed relatively stable neighbourhood population numbers during the study period. There was no evidence of a large change in population number in neighbourhoods where the suicidal behaviour clusters were identified (see Supplementary materials for more details).

### Comparison with previous studies

Our results showed that the spatio-temporal clusters of self-harm and suicide were fairly uncommon. Previous studies from Australia and the UK applying the scan statistics method also indicated the rarity of the phenomena – spatio-temporally cluster-related suicidal acts accounted for only 0–3% of total self-harm events and/or suicides (Cheung *et al.*, 2013; Hill *et al.*, 2020b; Jones *et al.*, 2013; Milner *et al.*, 2018; Too *et al.*, 2019, 2017; Too and Spittal, 2020). A few studies showed a higher proportion of suicides occurring in clusters. For example, Sy *et al.* (2019) found that 13.5% of suicides in 10 US states with the highest suicide rates were in clusters. However, the finding may be associated with the study’s relatively large time scale of aggregation (6 months) when the scan statistics method was applied. In other words, the study identified clusters of suicides that were already grouped (i.e., aggregated) every 6 months, and this may lead to an inflated number of suicides being identified in clusters. By contrast, the time scale of aggregation was 1 day for our study and the UK study (Jones *et al.*, 2013) and 1 month for Australian studies (Cheung *et al.*, 2013; Hill *et al.*, 2020b; Milner *et al.*, 2018; Too *et al.*, 2019, 2017; Too and Spittal, 2020). Another study from Spain indicated that 17.7% of suicides occurred in clusters (Perez-Costillas *et al.*, 2015); however, the study was based on a small sample (*n* = 96) from a defined rural area, making it difficult to compare its result to those of other much larger studies. In a recent study from Hong Kong, 19.2% of self-harm occurred in clusters (Leung *et al.*, 2018); however, this study used data from a highly densely populated area with many high-rise public housing buildings, which may contribute to the space–time concentration of self-harm episodes that occurred in residents who lived in different floors and had no contacts with one another.

In keeping with previous studies, our findings indicated that cluster-related suicidal acts were most likely to occur in males and younger people (Haw *et al.*, 2013; Hawton *et al.*, 2020). In a study from Wales, UK, suicide clusters were only identified in the subgroup of individuals aged 15–34 years but not in the all-age analysis (Jones *et al.*, 2013). The predominance of clustering of suicide in young people may reflect their greater susceptibility to identification with a model, which could be suicide by other young people or suicide by celebrities (Hawton *et al.*, 2020). Furthermore, we identified suicidal behaviour clusters in only age-specific analyses but not all-age analyses. The clustering of suicidal behaviour may be more likely to occur among people of a similar age rather than across age groups, as vulnerable individuals were more likely to identify with and be influenced by suicidal behaviour by people of the same age (Haw *et al.*, 2013). In a recent study of suicide clusters in young people in Australia, Hill *et al.* (2020a) found that 86% of suicides exposed to suicides of young people (aged 10–18 years; the index cases) belonged to the same age group as the index case. However, past research on sex and age patterns mainly focused on suicide clusters. Too *et al.* (2017) recently examined the characteristics of self-harm only clusters and did not find increased risk in any specific sex or age groups. Future research is needed to determine whether the sex and age patterns differ in self-harm and suicide clusters.

### Implications

Considering self-harm episodes and suicides together may identify more possible clusters of suicidal behaviour. Adopting advanced methodology such as the space–time permutation model and using data with fine-grained geographic information may allow identifying spatially exact clusters. Further research on the age and sex patterns of suicidal behaviour clusters and the transmission of suicidal behaviour within clusters can inform prevention and intervention plans.

## Data Availability

The data were provided by the governments of Taipei City and New Taipei City, Taiwan. The authors were not permitted to share the data.

## Supplementary material

For supplementary material accompanying this paper visit http://doi.org/10.1017/S2045796023000513. click here to view supplementary material
