# Supplementary material for: Space–time self-harm and suicide clusters in two cities in Taiwan
Source: Epidemiol Psychiatr Sci. 2023 Jun 1;32:e37. doi: 10.1017/S2045796023000513 (PMC10294245; doi:10.1017/S2045796023000513)

# Supplementary materials

1. Change in population number

In the analysis to detect the cluster of suicidal behavior using the space-time permutation scan statistics, if the population number of an area increases markedly within a short period of time in contrast to constant population in other areas, this could lead to a cluster of suicide or self-harm being detected in the area due to a rise in the underlying population and thus the number of suicide or self-harm despite no change in the incidence of suicide or self-harm. This may also occur when a new large neighborhood is developed. On the contrary, if the population number of an area decreases markedly within a short period of time in contrast to constant population in other areas, this could lead to a cluster of suicide or self-harm being missed in the area due to a drop in the underlying population and thus the number of suicide or self-harm despite a rise in the incidence of suicide or self-harm. Such population shift bias may occur when the study period is longer than a few years ([Kulldorff, 2018](#_ENREF_3)).

However, in our analysis, we set the maximum temporal size of the cluster at one year; this would limit the impact of change in the population number over time, if any. Furthermore, we checked neighborhoods’ changes in population during the study period and found largely small changes across neighborhoods. In Taipei City, the average annual change in population across the 432 neighborhoods was 0.49% (range -8.70%, 10.98%). In the 14 neighborhoods where clusters of suicidal behaviors were identified, the average annual change in population was 0.62% (range -0.65%, 3.08%). In New Taipei City, the average annual change in population across 1,032 neighborhoods was -0.19% (range -11.24%, 59.39%). In the 21 neighborhoods where clusters of suicidal behaviors were identified, the average annual change in population was -0.20% (range -1.17%, 4.73%). Last, our finding of the relatively small proportion of clustered suicide/self-harm detected in the space-time permutation models was consistent with that found in previous studies using Discrete Poisson model that could adjust for the population number ([Cheung *et al.*, 2013](#_ENREF_1); [Too *et al.*, 2017](#_ENREF_6); [Milner *et al.*, 2018](#_ENREF_4); [Too *et al.*, 2019](#_ENREF_5); [Hill *et al.*, 2020](#_ENREF_2); [Too and Spittal, 2020](#_ENREF_7)).

1. Identification of self-harm episodes and suicides

Supplementary Figure 1 shows the flowcharts to identify self-harm episodes and suicides for the study. Amongst the original 9,685 self-harm episodes for Taipei City and 33,787 episodes for New Taipei City, we excluded 3,740 and 12,765 episodes, respectively, that were ineligible for analysis due to an age <10 years, a residential address outside the study cities, with a fatal outcome (i.e., suicide), or duplicated records (for both cities), and additionally suicidal ideation, foreigners, or being reported to NSSS by non-hospital agencies (for New Taipei City only). We included self-harm hospital presentations only in the study for consistency. Among eligible self-harm episodes (n=5,945 for Taipei City and 21,022 for New Taipei City), events were further excluded if they had missing information on age, sex, or the date of self-harm or they had incomplete or incorrect addresses. A total of 5,291 and 20,531 self-harm records (89% and 98% of eligible episodes) in Taipei City and New Taipei City, respectively, were included in the analysis.

Amongst the original 1,428 and 2,052 suicides identified in Taipei City and New Taipei City, respectively, 14 and three with an age < 10 years were excluded, whilst an additional 395 suicides were identified from the national cause-of-death data files for New Taipei City. Of the 1,414 and 2,444 suicides eligible for analysis, eight and 115 were excluded due to incomplete addresses or missing information on sex or age, respectively. A total 1,406 (99% of eligible cases) and 2,329 (95%) suicides remained in the analysis for Taipei City and New Taipei City, respectively.

**References**

**Cheung YT, Spittal MJ, Williamson MK, Tung SJ and Pirkis J**. (2013) Application of scan statistics to detect suicide clusters in Australia. *PloS one* **8**, e54168.

**Hill NTM, Too LS, Spittal MJ and Robinson J**. (2020) Understanding the characteristics and mechanisms underlying suicide clusters in Australian youth: a comparison of cluster detection methods. *Epidemiology and Psychiatric Sciences* **29**, e151.

**Kulldorff M**. (2018) *SaTScan User Guide for version 9.6*. Available at <https://www.satscan.org/cgi-bin/satscan/register.pl/SaTScan_Users_Guide.pdf?todo=process_userguide_download>.

**Milner A, Too LS and Spittal M**. (2018) Cluster suicides among unemployed persons in Australia over the period 2001–2013. *Social Indicators Research* **137**, 189–201.

**Too LS, Pirkis J, Milner A, Robinson J and Spittal MJ**. (2019) Clusters of suicidal events among young people: do clusters from one time period predict later clusters? *Suicide & Life-Threatening Behavior* **49**, 561-571.

**Too LS, Pirkis J, Milner A and Spittal MJ**. (2017) Clusters of suicides and suicide attempts: detection, proximity and correlates. *Epidemiology and Psychiatric Sciences* **26**, 491-500.

**Too LS and Spittal MJ**. (2020) Suicide clusters among top 10 high-risk occupations: a study from 2001 to 2016 in Australia. *The Journal of Nervous and Mental Disease* **208**, 942-946.

## Supplementary Figure 1. Flow charts of identifying (a) self-harm episodes and (b) suicide deaths in Taipei City (2004-2006) and New Taipei City (2012-2016).

a)


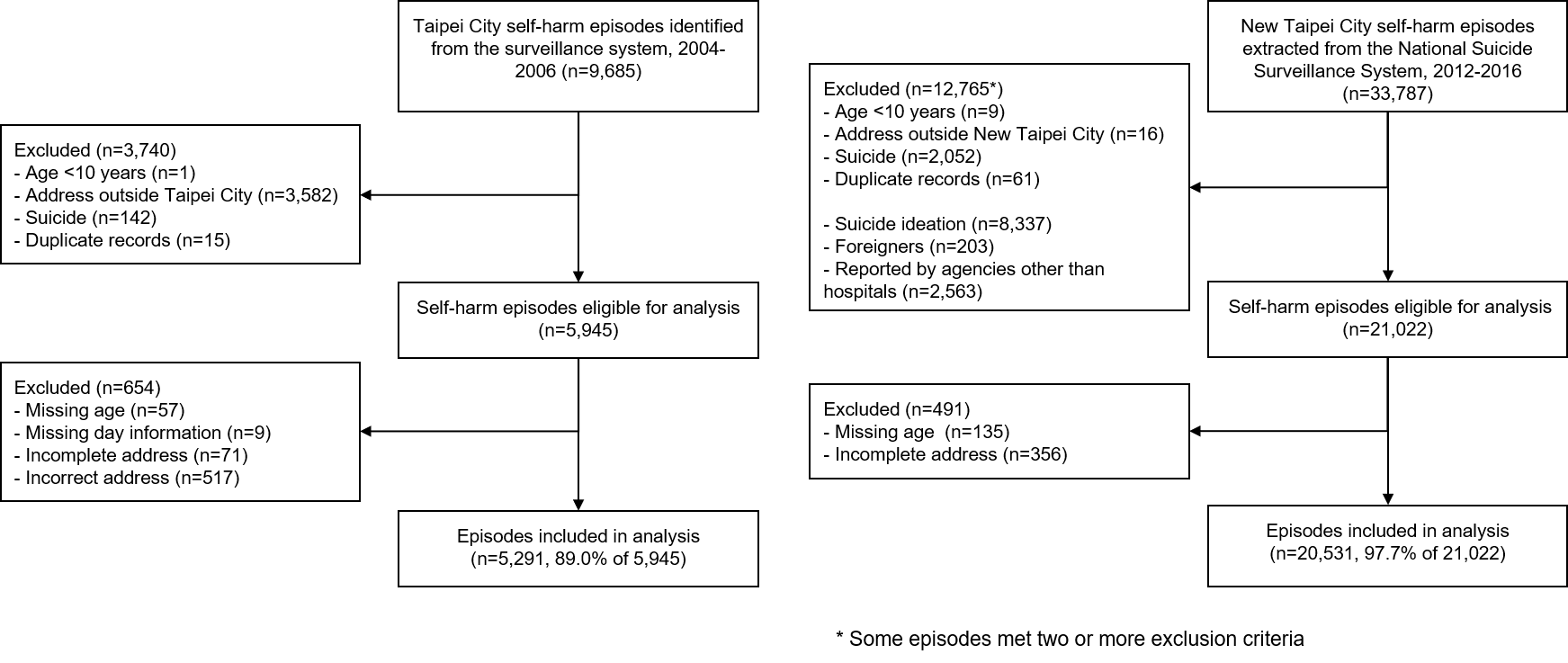


b)


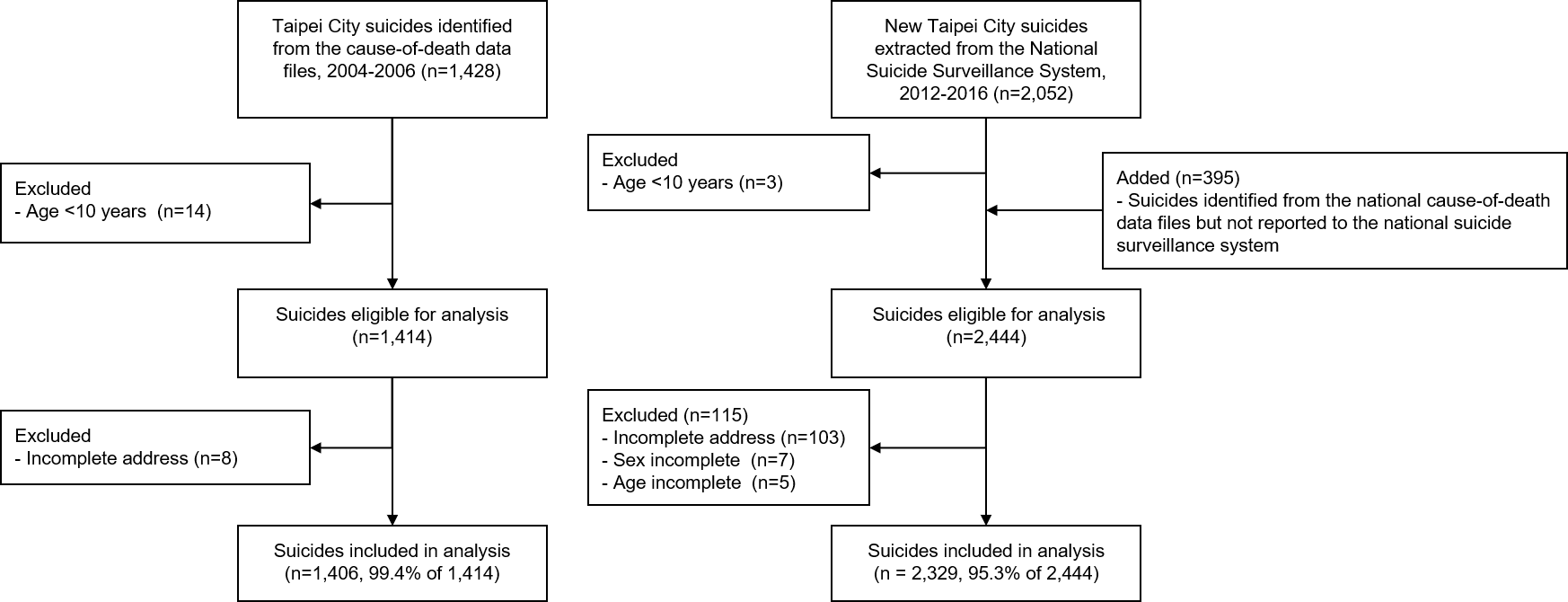

Supplement: Supplementary file 1 [file S2045796023000513sup001.docx]
